# Superinfections of the Spine: A Single-Institution Experience

**DOI:** 10.3390/jcm13102739

**Published:** 2024-05-07

**Authors:** Anthony K. Chiu, Bibhas Amatya, Idris Amin, Amit S. Ratanpal, Alexandra Baker Lutz, Brian M. Shear, Ivan B. Ye, Robin Fencel, Louis J. Bivona, Eugene Y. Koh, Julio J. Jauregui, Steven C. Ludwig, Daniel L. Cavanaugh

**Affiliations:** Department of Orthopaedics, University of Maryland School of Medicine, 110 S. Paca Street, 6th Floor Suite 300, Baltimore, MD 21201, USA

**Keywords:** superinfection, irrigation and debridement, reoperation, spine surgery

## Abstract

**Background/Objectives:** A superinfection occurs when a new, secondary organism colonizes an existing infection. Spine infections are associated with high patient morbidity and sometimes require multiple irrigations and debridements (I&Ds). When multiple I&Ds are required, the risk of complications increases. The purpose of this study was to report our experience with spine superinfections and determine which patients are typically affected. **Methods:** A retrospective case series of spine superinfections and a retrospective case–control analysis were conducted. Data were collected manually from electronic medical records. Spine I&Ds were identified. Groups were created for patients who had multiple I&Ds for (1) a recurrence of the same causative organism or (2) a superinfection with a novel organism. Preoperative demographic, clinical, and microbiologic data were compared between these two outcomes. A case series of superinfections with descriptive data was constructed. Lastly, two illustrative cases were provided in a narrative format. **Results:** A total of 92 patients were included in this analysis. Superinfections occurred after 6 out of the 92 (7%) initial I&Ds and were responsible for 6 out of the 24 (25%) repeat I&Ds. The preoperative erythrocyte sedimentation rate (ESR) and C-reactive protein (CRP) of the patients with a superinfection were significantly lower than those in the control group (*p* = 0.022 and *p* = 0.032). Otherwise, the observed differences in the preoperative variables were not statistically different. In the six cases of superinfection, the presence of high-risk comorbidities, a history of substance abuse, or a lack of social support were commonly observed. The superinfecting organisms included *Candida*, *Pseudomonas*, *Serratia*, *Klebsiella*, *Enterobacter*, and *Staphylococcus* species. **Conclusions:** Superinfections are a devastating complication requiring reoperation after initial spine I&D. Awareness of the possibility of superinfection and common patient archetypes can be helpful for clinicians and care teams. Future work is needed to examine how to identify, help predict, and prevent spine superinfections.

## 1. Introduction

Infections of the spine pose a significant risk to patients. They can occur postoperatively within a surgical wound or primarily through hematogenous seeding [1]. The leading causative agents are Gram-positive cocci, particularly *Staphylococcus aureus*, *Staphylococcus epidermidis*, and β-hemolytic *Streptococci* [2]. Gram-negative bacteria, such as *Klebsiella pneumoniae*, *Escherichia coli*, *Pseudomonas aeruginosa*, and *Proteus* species, are also frequently identified in postoperative wounds [3,4].

The initial treatment typically involves a surgical intervention of irrigation and debridement (I&D) with targeted antibiotic therapy [1]. In some instances, the initial treatment may be ineffective in eradicating the infection. In the case of a persistent infection, multiple I&Ds and broad-spectrum intravenous antibiotic regimens may be required [1,5]. One possible reason for the failure of the initial treatment is superinfection.

Superinfection occurs when a secondary infection develops in the setting of an existing infection [6]. It is a phenomenon most often studied in the context of immunodeficiency or pneumonia, but it can also be used to describe secondary wound infections [7]. A new, secondary microbial agent may colonize an existing wound infection from either external or internal sources [6]. Superinfection of the spine can complicate treatment, lead to multiple reoperations, cause significant morbidity for the patient, and cause a dramatic increase in the utilization of hospital resources [5]. For example, each additional I&D procedure carries a risk of additional blood loss, exposure to new pathogens, prolongation of antibiotic exposure, and anesthetic complications [8]. Finally, multiple I&D procedures add additional costs for the patient and increase their length of hospital stay, leading to medical complications like pneumonia and DVTs [9].

For suspected superinfections of the spine, it is first important to create a thorough differential diagnosis and screen for certain red flags [10,11]. For instance, post-procedure discitis could mimic superinfections, characterized by non-specific complaints of lower-back pain following surgical intervention [5,12]. Epidural abscesses should also be considered, especially if the patient demonstrates a neurological deficit [1]. Additionally, latent infections, including those instigated by organisms such as Propionibacterium acnes, warrant consideration, especially in the context of revision surgery [5].

It is also important to identify risk factors for post-surgical spinal infections. One common factor of note is diabetes mellitus (DM) [13,14]. The immune dysfunction caused by the effects of DM increases the probability of infection at the surgical site [13,14]. Appaduray and Lo demonstrated that patients with DM have an increased risk of repeated surgical site infections following lumbar spinal surgery [14,15]. In addition, fungal infections, particularly those caused by the *Aspergillus* species, must be considered in patients with diabetes as they lead to persistent or worsening infections postoperatively [16]. Furthermore, patients with spinal tuberculosis are highly susceptible to multiple deep postoperative spinal infections, adding significant morbidity [17].

To date, there have been no case reports or studies describing superinfection in the context of spine I&D procedures. The purpose of this study is to provide our single-institution experience with superinfections after spine I&D procedures. Our primary objective is to report the incidence of these superinfections, which is not currently known. In addition, we aim to report the demographics, risk factors, and microbiological characteristics of patients who developed superinfections after spine I&D procedures at our center.

## 2. Materials and Methods

After obtaining institutional review board (IRB) approval, a retrospective series and case–control analysis at a single academic institution was performed. The Strengthening The Reporting of Observational Studies in Epidemiology (STROBE) guidelines were used to ensure quality [18]. Patients who underwent I&D of the cervical, thoracic, or lumbar spine were identified by current procedural terminology (CPT) codes (10180, 22015, 22010) from July 2016 to August 2021. Adult patients aged between 18 and 88 years were included. Patients who underwent an initial surgery (prior to I&D) for an elective indication, trauma, known infection, or tumor were all included. Both primary and revision cases were included. On our initial screening, patients who did not undergo an I&D or who underwent deliberately staged I&Ds were excluded. This was performed to focus on unplanned reoperations only. In addition, some patients, upon screening, had no available culture data and were excluded. Data were collected manually from the patients’ electronic medical records.

For the patients who had undergone repeat I&D, their intraoperative culture results were reviewed from the first and second (repeat) I&D procedures. The patients were divided into three groups: (1) repeat I&D cultures showing the same organism, (2) a new organism, or (3) indeterminable. Cases showing a new organism on the repeat cultures after at least one week from the initial culture were deemed to have a superinfection. Cases were deemed indeterminable if the culture information for one of the I&Ds was not available, if the repeat cultures were taken over a short (<7 day) time span, if the new organisms on the repeat culture were thought to represent an initially polymicrobial infection, or if culture contamination was suspected.

The retrospective analysis compared the possible risk factors in the patients who had undergone repeat I&Ds. Specifically, the patients who had had a superinfection were compared to the patients who had undergone same-organism repeat I&D. The variables of interest were age, sex, operative diagnosis, infection location, duration of the first I&D, medical comorbidities, American Society of Anesthesiologists (ASA) physical status classification, length of hospital stay for the first I&D, presence of spinal hardware, social/substance use history, and the preoperative lab values prior to the first I&D (Table 1 and Table 2). The statistical analysis is further detailed in the “Statistical Methods” Subsection.

Next, a case series of patients who were re-infected with new organisms (those who had “superinfections”) was constructed. First, the demographic, clinical, and microbiologic data of these patients were tabulated. This key information was visualized in a set of summary tables for all the observed cases of superinfection (Table 3 and Table 4). Finally, two cases of superinfection were selected for narrative case presentations in order to illustrate the cadence of patient presentation and management.

### Statistical Methods

A univariate analysis was performed to compare the prevalence of the demographic and clinical risk factors for the patients who had undergone repeat I&D for a superinfection versus those who had undergone repeat I&D for the same organism. For the categorical variables, Chi-Squared tests or Fisher’s Exact tests were used where applicable. For the continuous variables, Welch’s modified two-sample t-test was used. The threshold for significance was set to an alpha of 0.05. All the statistical operations were performed using R software version 4.4.0 (R Core Team (2023), R Foundation for Statistical Computing, Vienna, Austria, 2023).

## 3. Results

### 3.1. Prevalence

**Figure 1:**
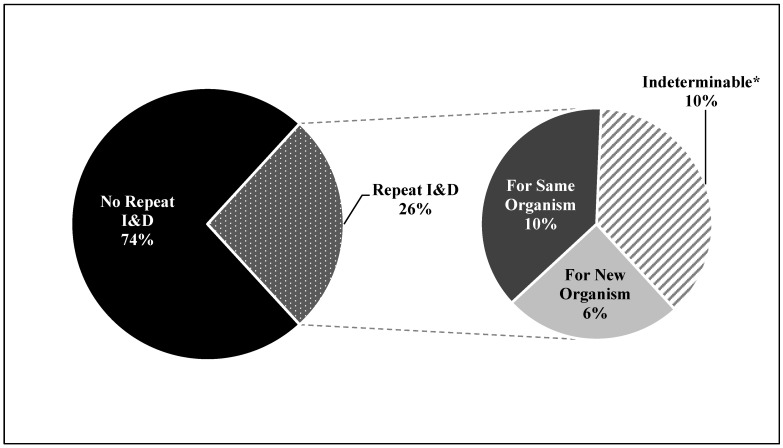
Prevalence of repeat irrigation and debridement for new organisms. * Indeterminable refers to patients who had undergone repeat I&D on the same admission and for which a superinfection could not be distinguished from an initially polymicrobial infection.

A total of 92 patients were identified who had undergone at least one I&D. Of these patients, 40 (43.5%) required I&D after an elective primary procedure, 29 (31.5%) required I&D after a revision procedure, 15 (16.3%) required I&D after a trauma-indicated procedure, 4 (4.3%) required I&D after a tumor-indicated procedure, and 8 (8.7%) required I&D for a known infection. There were 68 patients (73.9%) who had undergone a single I&D, and 24 (26.1%) who had required more than one I&D (Table 1). Among the patients who had required more than one I&D, there were nine patients whose cultures showed the same organism on the second I&D, six whose cultures showed a new organism on the second I&D, and nine whose cultures were indeterminable for the second I&D (Figure 1). Therefore, a total of 6 superinfections were identified, occurring in 6 out of the 92 (6.5%) patients who had required at least one I&D. Further, when looking only at the 24 patients who had required a second I&D, superinfection was responsible for 6 out of the 24 (25%) repeat I&Ds.

### 3.2. Case-Controlled Analysis

On average, the patients re-infected with a new organism on the repeat culture were older (average age 65.2 years) than the patients with the same organism on the repeat culture (55.0 years, *p* = 0.149). There were also more patients with diabetes in the new-organism group (*n* = 2, 33.3%) when compared to the same-organism group (*n* = 0, 0%, *p* = 0.143). The patients in the new-organism group had a shorter average hospital length of stay (LOS) (7.5 days) for the initial I&D when compared to the same-organism group (13.3 days, *p* = 0.058). In addition, the new-organism group had no preoperative leukocytosis on average (9.3), as opposed to the same-organism group, which did have preoperative leukocytosis on average (14.8, *p* = 0.166). Notably, the preoperative erythrocyte sedimentation rate (ESR) and C-reactive protein (CRP) of the patients in the new-organism group (ESR = 53.2, CRP = 5.8) were significantly lower than those in the same-organism group (ESR = 93.8, CRP = 20.1) according to the statistical analysis (*p* = 0.022 and *p* = 0.032, respectively) (Table 2).

There were no apparent differences in sex (*p* = 1), BMI (*p* = 0.244), operative diagnosis (*p* = 0.486 to *p* = 1), location (*p* = 0.525 to *p* = 1), duration of the first I&D (*p* = 0.742), ASA (*p* = 0.416), presence of spinal hardware (*p* = 0.417), substance abuse history (*p* = 0.400 to *p* = 1), preoperative lactate (*p* = 0.448), or preoperative glucose (0.561) (Table 2).

### 3.3. Case Presentation 1

A 57-year-old male with a past medical history of alcoholism, cirrhosis, and hepatitis C presented to the emergency department after being physically assaulted and sustaining a T4–5 fracture and dislocation injury. He underwent an uncomplicated C7–T7 posterior spinal instrumentation and fusion, 24 h of postoperative cefazolin, and was discharged in stable condition to a rehabilitation facility. A week later, the patient was readmitted for a superficial wound infection and underwent an I&D washout procedure, with cultures growing methicillin-resistant *Staphylococcus aureus* (MRSA). The patient was treated with vancomycin and dalbavancin, and he was discharged home. When the patient presented to the infectious disease clinic later that week, he was found to have persistent purulent drainage from his wound. He reported that he was not able to carry out dressing changes at home and did not have wound care support. The patient was brought back to the emergency department and was indicated for a repeat I&D. Gross purulence was found both superficial and deep to the fascia, and he underwent the planned I&D with cultures. His cultures revealed two new organisms: *Enterobacter hormaechei* and *Enterobacter cloacae*. The patient was treated with IV cefepime and IV ertapenem, and he was discharged to a rehabilitation facility on a 6-week course of ertapenem. He continued to experience a high drain output after surgery, which gradually diminished until complete resolution.

### 3.4. Case Presentation 2

A 62-year-old female with a past medical history of uncontrolled type 1 DM underwent elective L3–L5 lumbar decompression, instrumentation, and fusion. She was discharged to a rehab facility and initially progressed well. However, while at rehab, she developed an altered mental status (AMS) and grossly purulent urine. When she presented to the emergency department, she was in diabetic ketoacidosis (DKA) with a blood glucose level of 560 mg/dL. She developed urosepsis, bacteremia, and likely hematogenous seeding of the lumbar wound. After medical stabilization, I&D was indicated for persistent wound drainage. She underwent a first I&D with gross purulence noted, where her intraoperative cultures showed extended-spectrum beta lactamase (ESBL) *Klebsiella aerogenes*, *Klebsiella pneumoniae*, and methicillin-sensitive *Staphylococcus aureus*. She was treated with IV vancomycin and IV meropenem, and she was discharged on a 6-week course of ertapenem. However, one week later she presented, again, with an AMS. She was admitted to the intensive care unit in DKA for a second time, with an erythematous surgical wound and persistent purulent drainage. It was suspected that there were both non-compliance with her insulin regimen and up to a 3-day delay in antibiotic administration. She was deemed to have a recurrent wound infection with new osteomyelitis-discitis. She was initially treated with empiric IV vancomycin and IV meropenem until medically stabilized. Once she was stable, she returned to the OR for repeat I&D and staged reconstruction, including the removal of hardware, revision L3-Pelvis instrumentation, and partial corpectomies at L4 and L5. Repeat cultures revealed *Candida albicans*, *Candida dubliniensis*, *Klebsiella aerogenes*, and MRSA.

### 3.5. Case Series

There were six cases of superinfection identified. In the six cases of superinfection, the presence of high-risk comorbidities, a history of substance abuse, or a lack of social support were commonly observed. The superinfecting organisms included *Candida*, *Pseudomonas*, *Serratia*, *Klebsiella*, *Enterobacter*, and *Staphylococcus* species. Further clinical and microbiologic details of the superinfection cases are presented in Table 3 and Table 4.

## 4. Discussion

Recurrent spinal infections requiring multiple I&Ds are detrimental to patients and the healthcare system. This case series described superinfection with new organisms as a contributor to patient morbidity and a possible culprit for hospital readmission and reoperation.

Of all the I&Ds in the current study, approximately 7% (6/92) eventually resulted in superinfection. In addition, at least one quarter (25%) of the repeated I&Ds produced cultures consistent with superinfection. This case series is the first in the literature to provide a focused description of superinfection in the postoperative period after spine surgery. Our findings are consistent with studies in different subspecialties. In the context of arthroplasty, Darwich et al. found that 23.7% of 169 periprosthetic joint infections (PJIs) eventually evolved into superinfections with a new organism. The authors then showed that patients with superinfections had worse outcomes in terms of infection eradication, therapy failure, number of revisions, and mortality [19]. While our series showed a lower rate of superinfection than Darwich et al.’s, we deemed nine cases as indeterminable. Therefore, the true rate of superinfection after spine I&D could be higher than what has been reported in our series.

The causes of superinfection are likely multifactorial. Based on our review, patient factors such as poor medical literacy, non-compliance, or being relatively immunocompromised (due to age, comorbidities, medications, or substance abuse) may be contributing factors. Further, a lack of social support such as transportation barriers, poor nutrition, or poor postoperative wound care may complicate treatment. Lastly, surgical factors, antibiotic regimen, and microbial properties may also play a role.

In our study, the patients with superinfections tended to be older on average, with medical comorbidities making them relatively immunocompromised. Notable comorbidities included uncontrolled DM, polycythemia vera, rheumatoid arthritis, liver cirrhosis, chronic kidney disease, and malnutrition. Furthermore, these patients often had a history of substance abuse, including alcohol, marijuana, tobacco, and cocaine. It is well established that immunocompromised states and substance abuse are risk factors for postoperative wound infection in the spine [20,21]. Therefore, their presence in the patients in our study who developed superinfection was not surprising. Prior to elective spine surgery, careful clinical consideration and shared decision making must be employed for these higher-risk patients, knowing that both an initial infection and a superinfection could complicate the postoperative course.

Interestingly, the patients who eventually developed superinfections had less elevated preoperative ESR and CRP before their first I&D. This could be explained by differences in their immune system health. For example, in the literature on septic arthritis, it has been found that immunocompromised patients had insignificant increases in their ESR and CRP in response to culture-positive infection [22]. Despite this, other studies suggest that, in septic arthritis, ESR and CRP have no difference in their diagnostic utility between immunocompetent and immunocompromised patients [23]. Unfortunately, there are no comparable studies available that investigate ESR and CRP levels as risk factors for superinfection in the spine. Another interesting finding in our study was that the patients who had undergone repeat I&D for superinfections had a shorter average LOS after their initial I&D than the patients who had undergone repeat I&D for the same organism. This was not found to be statistically significant and, therefore, must be interpreted with skepticism. Regardless, the length of stay of a patient undergoing I&D should be carefully considered within the full clinical picture, with the awareness that the risk for superinfection is potentially elevated with an earlier discharge. While a shorter LOS decreases the healthcare burden, the occurrence of a superinfection increases the healthcare burden through antibiotic resistance and the need for broader-spectrum therapy.

Antibiotic resistance is another factor that may play a role in spine superinfections. In 2019, the WHO identified antimicrobial resistance as one of the top 10 threats to global health [24]. Priority status was given to the ESKAPE pathogens (*Enterococcus faecium*, *Staphylococcus aureus*, *Klebsiella pneumoniae*, *Acinetobacter baumannii*, *Pseudomonas aeruginosa*, and *Enterobacter* species) [25]. In addition, another category of problematic pathogens is represented by “difficult-to-treat” (DTT) organisms, which are defined by a resistance to biofilm-active antibiotics. Studies focusing on PJI have revealed that superinfection with DTT organisms is associated with worse outcomes than superinfection with non-DTT organisms [26]. In our sample, the susceptibility studies showed that many of the superinfecting organisms exhibited antibiotic resistance, including fluconazole-resistant *Candida*, ampC-producing *Serratia*, and multidrug-resistant *Klebsiella*. Two of the six cases were primarily infected with ESKAPE organisms and were later superinfected by the Candida species. Another two of the six cases were primarily infected with MRSA and went on to be superinfected by other ESKAPE organisms. In addition, two of the six superinfections in our series were considered DTT. Further reports of superinfection may help to determine if our observation of superinfection after spine I&D was patient-specific or part of a global problem of antibiotic resistance.

## 5. Limitations

This study had several limitations. Due to its retrospective nature, there was the opportunity for selection bias and variable quality of the data from the electronic medical records. However, given the nature of our topic, the current study design was considered the most appropriate option. In addition, this study population was derived from an academic tertiary referral center that treats complex patients, which may limit the generalizability of our findings to other healthcare settings. Finally, our underpowered sample size limited our ability to capture statistically significant differences, and large-scale registry data may be required to determine the true risk of many of the preoperative comorbidities.

## 6. Conclusions

Awareness of the possible risk of superinfection and common patient archetypes can be helpful for clinicians and care teams. Superinfections carry substantial morbidity to the patient and burden the healthcare system. Further dialogue may help reduce the occurrence of spine superinfections and identify possible root causes.

## Figures and Tables

**Table 1:** Overview of the patient sample.

Patient Variable	Number of Patients (%)
Total Patients Who Underwent I&D	92
Index Procedure Type (Prior to I&D) *	
Primary Elective	40 (43.5%) → 3 superinfections
Revision	29 (31.5%) → 2 superinfections
Trauma	15 (16.3%) → 1 superinfection
Tumor	4 (4.3%) → 0 superinfections
Known Infection	8 (8.7%) → 1 superinfection
Number of I&Ds Required	
Single I&D	68 (73.9%)
Multiple/Repeat I&Ds	24 (26.1%)
Microbiology of Multiple/Repeat I&Ds	
Same Organism	9 out of 24 (37.5%)
New Organism (“Superinfection”)	6 out of 24 (25.0%)
Indeterminable	9 out of 24 (37.5%)
Overall Repeat I&Ds for Superinfections	6 out of 92 (6.5%)

I&D: irrigation and debridement. * One case of superinfection was both an index revision surgery and a known infection case. The total count was 6 for all superinfections.

**Table 2:** Case-controlled analysis of repeat irrigations and debridements for new organisms (“Superinfections”).

Characteristic	New Organism (“Superinfection”)	Same Organism	*p* Value
Total	6	9	
Age			
Average ± SD	65.2 ± 12.0	55.0 ± 13.3	0.149
18–29	0 (0%)	0 (0%)	
30–49	1 (16.7%)	3 (33.3%)	
50–69	3 (50.0%)	4 (44.4%)	
70+	2 (33.3%)	2 (22.2%)	
Sex			
Male	3 (50.0%)	5 (55.6%)	1
Female	3 (50.0%)	4 (44.4%)	1
BMI			
Average ± SD	28.5 ± 4.3	32.3 ± 7.7	0.244
Procedure Diagnosis			
Postoperative Wound Infection	5 (83.3%)	7 (77.8%)	1
Epidural or Subdural Abscess	0 (0%)	2 (22.2%)	0.486
Paraspinal Abscess	0 (0%)	1 (11.1%)	1
Osteomyelitis-Discitis	1 (16.7%)	2 (22.2%)	1
Pseudo-meningocele	1 (16.7%)	1 (11.1%)	1
Esophageal Perforation	0 (0%)	0 (0%)	
Location			
Cervical	2 (33.3%)	1 (11.1%)	0.525
Thoracic	2 (33.3%)	3 (33.3%)	1
Lumbar	2 (33.3%)	5 (55.6%)	1
Duration of 1st I&D			
Average ± SD	2.3 ± 1	2.1 ± 1.3	0.742
Medical History			
DM	2 (33.3%)	0 (0%)	0.143
Renal Disease	0 (0%)	1 (11.1%)	1
Autoimmune Disease	1 (16.7%)	0 (0%)	0.400
Psychiatric History	5 (83.3%)	4 (44.4%)	0.287
Hepatitis B Virus Infection	1 (16.7%)	0 (0%)	0.400
Hepatitis C Virus Infection	1 (16.7%)	2 (22.2%)	1
Human Immunodeficiency Virus Infection	0 (0%)	0 (0%)	
Use of Steroids	2 (33.3%)	1 (11.1%)	0.525
ASA	3.0 ± 0	2.8 ± 0.7	0.416
LOS	7.5 ± 2.4	13.3 ± 7.6	0.058
Prior Spinal Hardware Present	5 (83.3%)	7 (77.8%)	0.417
Social History			
Alcohol Abuse	1 (16.7%)	0 (0%)	0.400
Current Smoker	2 (33.3%)	3 (33.3%)	1
Former Smoker	2 (33.3%)	1 (11.1%)	0.525
IVDU	0 (0%)	2 (22.2%)	0.486
Labs			
White Blood Cells	9.3 ± 6.1	14.8 ± 8.4	0.166
C-Reactive Protein	5.8 ± 4.7	20.1 ± 14.8	**0.022**
Erythrocyte Sedimentation Rate	53.2 ± 23.5	93.8 ± 41.5	**0.032**
Lactate	2.6 ± 2.3	1.8 ± 0.9	0.448
Glucose	217 ± 321.6	134.6 ± 60.8	0.5611

SD, standard deviation; BMI, body mass index; DM, diabetes mellitus; ASA, American Society of Anesthesiologists physical status classification system; LOS, length of stay; and IVDU, intravenous drug use. Bolded values represent p-values less than 0.05.

**Table 3:** Demographics and clinical data. * Cases with superinfection.

Case	Age, Sex	Race	BMI	ASA	Medical Comorbidities	Substance History	ESR	CRP	WBCs	Lactate
1	49, F	White	34	3	Polycythemia vera, multiple preoperative UTIs, urinary incontinence, poor dentition, anxiety, depression Albumin dropped to the 2.2–2.8 range (low) postoperatively Diagnosed with liver cirrhosis + esophageal varices and severe protein-calorie malnutrition shortly after first I&D	Tobacco, Marijuana	85	3.7	3.5	--
2	64, M	White	24	3	Anxiety, depression, hypertension, urinary retention	Tobacco, Marijuana, Cocaine	22	2	12.2	--
3	62, F	Black	32	3	Uncontrolled type 1 DM, depression, CAD, HTN, HLD, obesity Presented with DKA and UTI	Tobacco	--	--	19.2	5.1
4	79, M	White	27	3	Uncontrolled type 2 DM, colon cancer s/p chemotherapy, HTN, HLD, CAD, carotid stenosis, TIA, GERD	Tobacco	41	13.8	6.8	0.8
5	57, M	White	31	3	Alcoholic liver cirrhosis, chronic hepatitis C, cleared hepatitis B, atrial fibrillation, CHF, HTN, HLD *MRSA* cellulitis in bilateral lower extremities thought to be the infectious source	Alcohol Abuse	58	5.5	3.1	1.8
6	80, F	White	24	3	Rheumatoid arthritis in immunosuppression (hydroxychloroquine, leflunomide, prednisone), hypothyroidism, adrenal insufficiency, CKD III/IV, anxiety, HTN, HLD, asthma, diverticulitis, GERD, gout	None	60	3.8	10.9	--

F, female; M, male; BMI, body mass index; ASA, American Society of Anesthesiologists physical status classification system; UTI, urinary tract infection; I&D, irrigation and debridement; DM, diabetes mellitus; CAD, coronary artery disease; HTN, hypertension; HLD, hyperlipidemia; DKA, diabetic ketoacidosis; TIA, transient ischemic attack; GERD, gastroesophageal reflux disease; CHF, congestive heart failure; MRSA, methicillin-resistant *Staphylococcus aureus*; CKD, chronic kidney disease; ESR, erythrocyte sedimentation rate; CRP, C-reactive protein; and WBCs, white blood cells. * Based on the most recent preoperative data or information.

**Table 4:** Microbiologic data: cases with superinfection.

Case	Age, Sex	Level	Diagnosis	Initial Organism	Length of I&D (Hours)	Antibiotics *	LOS * (Days)	Weeks to Repeat I&D	Superinfecting Organism
1	49, F	L4-Pelvis	Osteomyelitis, Sacroiliitis	*Coagulase-negative Staphylococci*	2.3	IV daptomycin and ceftriaxone in hospital Discharged on daptomycin and rifampin	7	9	*Candida albicans*, *Candida glabrata*
2	64, M	C4-C7	Postoperative Wound Infection	*Propionibacterium acnes*, *Enterococcus faecalis*, *Staphylococcus hominis*	1.4	IV vancomycin and piperacillin-tazobactam in hospital Discharged on ampicillin and rifampin	5	12	*Serratia marcescens*
3	62, F	L3-L5	Postoperative Wound Infection	*Klebsiella aerogenes*, *Klebsiella pneumoniae*, *MSSA*	1.9	IV vancomycin and meropenem in hospital Discharged on vancomycin and ertapenem	12	3	*Candida albicans*, *Candida dubliniensis*, *Klebsiella aerogenes*, *MRSA*
4	79, M	T11-L3	Postoperative Wound Infection, Pseudo-meningocele	*MRSA*	1.7	IV vancomycin, piperacillin-tazobactam, and rifampin in hospital Discharged on vancomycin and rifampin	8	3	*Serratia marcescens*
5	57, M	C7-T7	Postoperative Wound Infection	*MRSA*	2.1	IV vancomycin and dalbavancin in hospital Discharged with repeat dalbavancin, outpatient	6	2	*Enterobacter hormaechei*, *Enterobacter cloacae*
6	80, F	Occiput-T2	Postoperative Wound Infection	*MRSA*	4.1	IV daptomycin in hospital Discharged on daptomycin and rifabutin	7	7	*Pseudomonas aeruginosa*, *Staphylococcus caprae*

F, female; M, male; I&D, irrigation and debridement; MRSA, methicillin-resistant *Staphylococcus aureus*; IV, intravenous; and LOS, length of hospital stay. * Referring to the admission for the index I&D procedure.

## Data Availability

The datasets presented in this article are not readily available because public data sharing has not been approved by the local institutional review board. Requests to access the datasets should be directed to the corresponding author, and will be assessed pending institutional review board approval.
